# Dosimetric study of the plan quality and dose to organs at risk on tangential breast treatments using the Halcyon linac

**DOI:** 10.1002/acm2.12655

**Published:** 2019-06-11

**Authors:** Everardo Flores‐Martinez, Gwe‐Ya Kim, Catheryn M. Yashar, Laura I. Cerviño

**Affiliations:** ^1^ Department of Radiation Medicine and Applied Sciences University of California San Diego San Diego CA USA

**Keywords:** breast, Dose to OARs, Halcyon, IGRT

## Abstract

**Purpose:**

To investigate the plan quality and doses to the heart, contralateral breast (CB), ipsilateral lung (IL), and contralateral lung (CL) in tangential breast treatments using the Halcyon linac with megavoltage setup fields.

**Methods:**

Radiotherapy treatment plans with tangential beams from 25 breast cancer patients previously treated on a C‐arm linac were replanned for Halcyon. Thirteen corresponded to right‐sided breasts and 12 to left‐sided breasts, all with a dose prescription of 50 Gy in 25 fractions. Plans were created with the following setup imaging techniques: low‐dose (LD) MVCBCT, high‐quality (HQ) MVCBCT, LD‐MV and HQ‐MV pairs and the imaging dose was included in the plans. Plan quality metric values for the lumpectomy cavity, whole‐breast and doses to the organs at risk (OARs) were measured and compared with those from the original plans.

**Results:**

No significant differences in plan quality were observed between the original and Halcyon plans. An increase in the mean dose (Mean) for all the organs was observed for the Halcyon plans. For right‐sided plans, the accumulated Mean over the 25 fractions in the C‐arm plans was 0.4 ± 0.3, 0.2 ± 0.2, 5.4 ± 1.3, and 0.1 ± 0.1 Gy for the heart, CB, IL, and CL, respectively, while values in the MVCBCT‐LD Halcyon plans were 1.2 ± 0.2, 0.6 ± 0.1, 6.5 ± 1.4, and 0.4 ± 0.1 Gy, respectively. For left‐sided treatments, Mean in the original plans was 0.9 ± 0.2, 0.1 ± 0.0, 4.2 ± 1.2, and 0.0 ± 0.0 Gy, while for the MVCBCT‐LD Halcyon plans values were 1.9 ± 0.2, 0.6 ± 0.2, 5.1 ± 1.2, and 0.5 ± 0.2 Gy, respectively.

**Conclusions:**

Plan quality for breast treatments using Halcyon is similar to the quality for a 6 MV, C‐arm plan. For treatments using megavoltage setup fields, the dose contribution to OARs from the imaging fields can be equal or higher than the dose from treatment fields.

## 1 INTRODUCTION

### 1.1 Breast cancer

Breast cancer is the cancer with the highest incidence in women in the US after skin cancer.^1^ The American Cancer Society estimated 266 120 new cases in women in 2018, which was about 30% of the new female cancer cases, excluding skin cancer.^2^ During 2010–2014, in the US, the median age at diagnosis for breast cancer patients was 62 yr.^3^ Breast cancer treatment options depend on the stage of the disease, and include mastectomy, breast conserving surgery (BCS), chemotherapy, and radiation. For stages I and II, approximately 60% of the treated patients undergo radiation therapy,^3^ which may be administered as external beam therapy, brachytherapy, or a combination of them.

### 1.2 IGRT

For patients receiving radiation therapy, image‐guided radiotherapy (IGRT) is used to provide an accurate setup at the treatment machine ensuring adequate coverage of the target volume. Different imaging techniques have been used for IGRT, such as orthogonal kilovoltage (kV) or megavoltage (MV) pairs, kV or MV cone beam computed tomography (CBCT), ultrasound, surface‐guided radiotherapy (SGRT), or magnetic resonance imaging. For MV‐based imaging techniques, the dose from the imaging fields to organs at risk (OARs) can be significant, and needs to be evaluated. The American Association of Physicists in Medicine (AAPM) Task Group 75 recommended that the total dose should be evaluated patient‐by‐patient, assessing individual exposure risk and also recommended to optimize the imaging protocols to decrease the dose to OARs.^4^ For breast cancer patients, reducing the dose to OARs from radiotherapy treatments is important as the 10‐year survival probability for diagnosed stage I and II patients treated with radiation after breast‐conserving therapy is higher than 60%.^5^ Several studies have reported dose to the OARs from the different modalities available for radiation delivery including three dimensional conformal therapy (3D‐CRT),^6^, ^7^ tomotherapy,^8^, ^9^, ^10^, ^11^, ^12^, ^13^ static‐field intensity‐modulated radiation therapy (IMRT)^10^, ^12^, ^14^, ^15^ and volumetric arc therapy (VMAT).^8^, ^10^, ^15^, ^16^, ^17^, ^18^

### 1.3 Halcyon

The Halcyon^TM^ system is a bore‐enclosed, jawless linac using a 6 MV flattening‐filter free (FFF) beam, where beam modulation is achieved by means of a stacked and staggered dual‐layer multi‐leaf collimator (MLC). Halcyon treatments are 100% image‐guided, allowing a faster turnaround due to its high efficiency while ensuring accurate setup. Another distinctive feature of the Halcyon is that it does not have light field, optical distance indicator or lasers at the treatment isocenter. Instead, it has a virtual isocenter out of the bore identifiable by external lasers. In a typical treatment, the patient is setup using the virtual isocenter lasers, and then the couch is moved into the bore to the treatment isocenter and patient setup is verified through imaging. For the version 1.0 of Halcyon, all the imaging setup fields are taken using the same 6 MV FFF beam used for treatment and the Digital Megavoltage Imaging (DMI) panel. When a Halcyon plan is created the user has the option to select among four different setup fields:

- MVCBCT HQ. High‐quality megavoltage cone beam CT, delivering 10 monitor units (MUs) as the gantry rotates clockwise from 260° to 100°.
- MVCBCT LD. Low‐dose MVCBCT, delivering 5 MUs in a clockwise gantry rotation from 260° to 100°.
- MV‐MV HQ. High‐quality orthogonal MV radiograph pair, with one image acquired with the gantry at 0° and the second one at 90°. Two MUs are delivered for each field.
- MV‐MV LD. Low‐dose MV orthogonal radiograph pair, delivering one MU per image.

All the setup fields are delivered with the collimator fixed at 0° and the imaging field size is set by the user. For the MVCBCT, the size on the X axis is fixed to 28.0 cm and in the Y direction the field size can go from 2.0 to 28.0 cm on 2.0 cm increments. For the MV‐MV fields, the aperture in the X axis can go from 2.0 to 28.0 cm in 0.1 cm increments, while for the Y axis, the aperture can take values from 2.0 to 28.0 on 2.0 cm increments. Dose from the imaging setup fields is accounted for during plan optimization and reported as part of the total dose in the plan.

This work presents an evaluation of the plan quality and dose metrics to OARs for left‐ and right‐sided breast plans using Halcyon, and compares the results with metrics obtained for plans created for a C‐arm linac. The relative contribution of imaging dose to OARs was calculated, and dose reduction techniques are suggested.

## 2 METHODS

### 2.1 Treatment planning

#### 2.1.1 Plan selection

This study was performed for 25 breast plans, 12 left‐sided, and 13 right‐sided, corresponding to patients with stages I and II, without nodal involvement, undergoing radiation after breast conservative surgery. Patients had a chest wall separation of less than 22 cm so that 6 MV beams were appropriate for planning. The dose prescription was 50 Gy in 25 fractions using tangential 6 MV photon beams and a boost of 10 Gy in 5 fractions with electron beams (6 or 9 MeV). Patients were treated with a Varian 21EX linac with a Millennium 120 MLC (Varian Medical Systems, Palo Alto, CA, USA). The tangential photon plans were replanned for the Halcyon system to compare plan quality and dose to OARs.

#### 2.1.2 Simulation and contouring

Patient simulation was performed with a GE CT scanner available at our radiotherapy department. The slice thickness was 2.5 mm and the scans covered from the mid‐abdomen to the clavicle with the patients laying on a 10°–20° inclined breast board in the supine position with both arms raised. The image series were imported into the Eclipse treatment planning system (Varian Medical Systems, Palo Alto, CA, USA) for contouring and planning. The physician contoured the lumpectomy cavity and reviewed the contours for OARs: ipsilateral lung (IL), contralateral lung (CL), and heart. For this study, treatment breast and contralateral breast (CB) were also contoured including all the palpable breast and setting the borders to exclude the chest wall and 5 mm from the skin. The medial border was set to the mid‐sternum and the caudal limit to 2 cm below the inframammary line.

#### 2.1.3 C‐arm planning

For the original plans, using the 21EX linac, the tangential fields were defined using the linac jaws. The superior limit was set to the head of the clavicle and the inferior limit to 2 cm below the inframammary line, the medial margin was set to the mid sternum, and the anterior limit included a 2 cm flash from the contour of the body. Gantry angles were set such that the posterior edges of the opposing fields were coplanar and preventing the fields to extend more than 2 cm into the lungs. Dose was calculated with a grid size of 0.25 cm using the Anisotropic Analytical Algorithm (AAA), version 13.6. The irregular surface compensator tool was used for both fields, and hot spots larger than 105% were cleared by using the fluence editor available in Eclipse. For left‐sided treatments, the heart was spared by adding a heart block defined by the MLC.

#### 2.1.4 Halcyon planning

**Figure 1:**
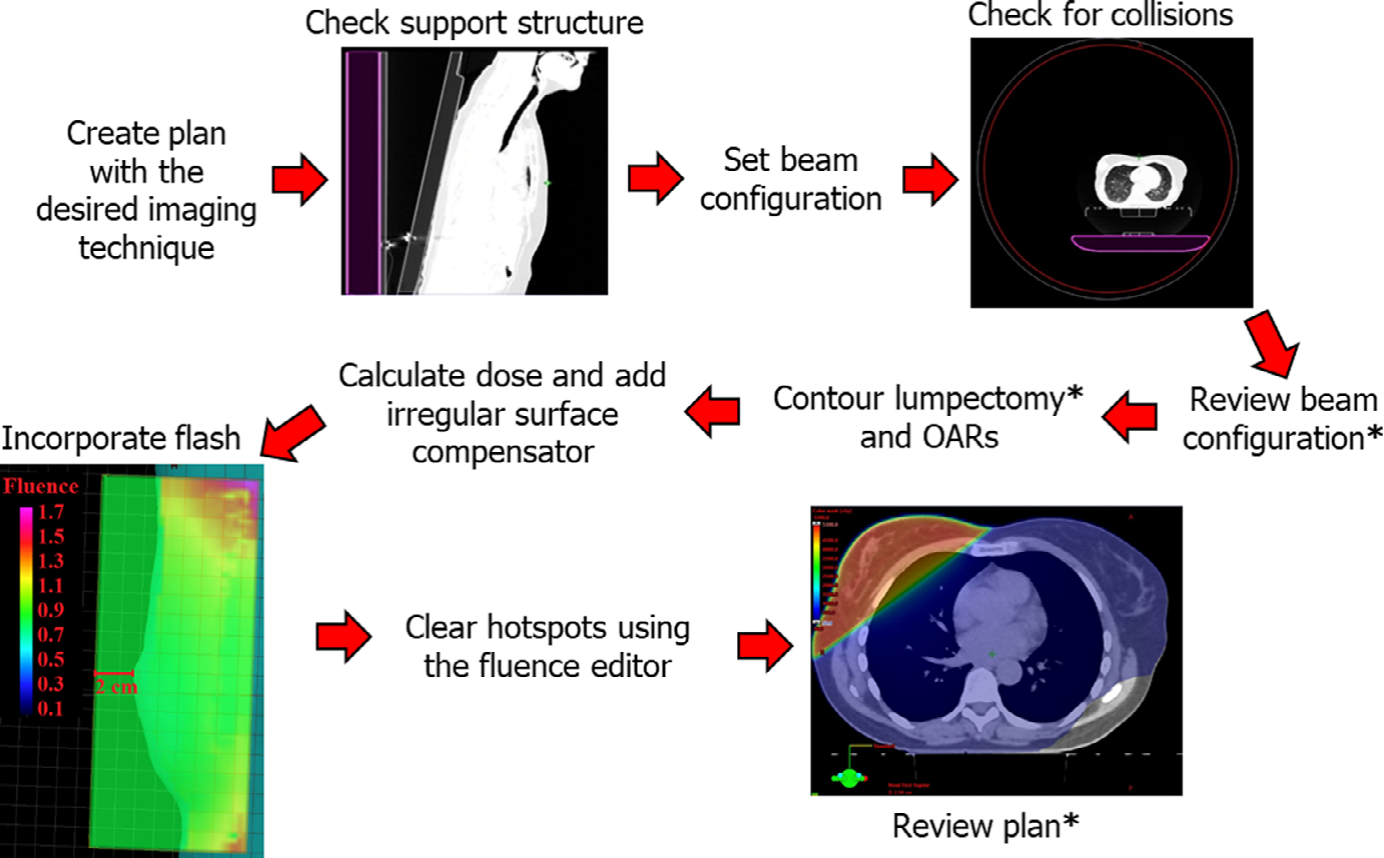
Workflow for tangential whole breast treatments in the Halcyon^TM^ system, steps performed by the Radiation Oncologist is indicated with an asterix.

Figure 1 shows the workflow we used to generate tangential breast treatments with Halcyon. The planning process starts by selecting one of the imaging techniques available and the field size for the imaging beams. The dose contribution from these imaging fields will be included in the total dose reported by the treatment planning system. The second step is to check and correct the positioning of the couch. When a plan is created for Halcyon, the couch is positioned automatically by Eclipse but when the breast board is used, the automatic positioning of the couch may be inaccurate. This task is performed in the axial view and verified on the sagittal view using a lung window leveling as seen in Fig. 1. The next step is to define the isocenter, which can be moved directly on the axial view. A constraint for Halcyon is that the position of the isocenter is limited by the lateral range of the couch inside of the bore. This poses a challenge when planning for larger patients, as the isocenter would need to be displaced more laterally. If the position of the isocenter is such that the couch collides with the bore, the bore will appear highlighted on the axial view and the system will not perform any dose calculation. Once the isocenter is set without collisions, the beam angle can be adjusted on the axial view and the collimator angle value can be entered manually while checking it on the beam’s eye view with the body contour turned on. The field sizes are adjusted on the beam’s eye view, setting the superior limit to the head of the clavicle and the inferior limit to 2 cm below the inframammary line, the medial margin to the mid sternum, and the anterior limit including a flash from the contour of the body. The skin flash will be adjusted on a later step. A similar approach is done for the opposing field, ensuring that gantry angles are set such that the posterior edges of the opposing fields are coplanar and preventing the fields to extend more than 2 cm into the lungs. At our clinic, the beam configuration is checked and modified if needed by the radiation oncologist to ensure appropriate coverage. The oncologist provides the contouring for the lumpectomy and other OARs are contoured as well. The first dose calculation is performed with the initial beam configuration and then, the irregular surface compensator is inserted for each beam with a penetration depth of 50%. At this point the flash is incorporated, by measuring the transmission factor of a point near the edge of the body contour and assigning that value (usually between 0.75 and 0.90) to the surrounding area 2 cm beyond the most anterior point of the body. Finally, the fluence editor is used to reduce hotspots larger than 105% of the prescription dose and the plans are reviewed by the radiation oncologist, who checks the coverage, normalization, and dose to OARs.

In this work, four Halcyon plans, one for every imaging technique available were created for each patient.

The Halcyon plans had the same isocenter and beam configuration as the C‐arm plans and the tangential fields were defined using the MLC with the same aperture as the C‐arm plans. The field sizes for the imaging fields were (14.0 × 10.0) cm^2^ and (14.0 × 14.0) cm^2^ for the MVCBCT and MV pairs, respectively. Dose was calculated with a grid size of 0.25 cm using the AAA, version 15.1. To investigate the contribution to the total dose from the imaging fields, each Halcyon plan was duplicated and the treatment fields were erased.

### 2.2 Plan quality evaluation

The total number of monitor units (MUs) was compared for C‐arm and Halcyon plans. Dose volume histogram (DVH) metrics naming followed TG‐263 guidelines^19^ and were calculated using the visual scripting tool in Eclipse. Coverage of the lumpectomy cavity and the whole breast were evaluated by using the mean dose (Mean), and D98%[%], the minimum dose received by the hottest 98% subvolume. The homogeneity index was calculated according with the ICRU83 recommendations:^20^

$$\text{HI =}\;\frac{\text{D2[\textbackslash{}\% ] - D98[\textbackslash{}\% ]}}{\text{D50[\textbackslash{}\% ]}}$$

and the prevalence of hot spots was evaluated measuring V105%[%], the hottest subvolume receiving more than 105% of the prescribed dose. Dose volume histogram metrics for the lumpectomy and whole breast were compared grouping together right‐sided and left‐sided treatments, while doses the OARs were assessed measuring volumes receiving relevant doses as well as point maximum doses (Max) and Mean. Organs at risks DVH parameters were evaluated separately for right‐sided and left‐sided treatments.

Statistical analysis was performed with Origin 9.6.5 (OriginLab Corporation, Northampton, MA, USA), comparing each parameter of the C‐arm and Halcyon plans using the nonparametric, two‐sample Kolmogorov‐Smirnov test. Significant difference was considered for *P* < 0.01.

## 3 RESULTS

### 3.1 Plan quality

Halcyon plans showed an average 35% increase in the number of monitor units per fraction as compared with the C‐arm plans (*P* < 0.001). This increase can be attributed to the use of the 6X FFF beam in Halcyon as compared with the 6 MV flattened beam delivered by the C‐arm linac. We observed that this increment was higher in cases where the isocenter was not positioned at the center of the breast. Table 1 shows the plan quality comparison of the C‐arm and Halcyon plans. For the lumpectomy, none of the DVH metrics investigation showed a significant difference (at the 0.01 level). For the whole breast, no significant difference was found for Max, Mean, and V105% [%]. The values of D98%[Gy] and H.I. for the Halcyon plans showed a statistically significant difference as compared with the C‐arm plans, however, given that those differences are less than 1%, we do not consider them of clinical relevance and could be related with the planner’s skill when editing fluence.

**Table 1:** Lumpectomy and whole breast DVH metrics comparison between C‐arm and Halcyon plans Stated values indicate mean ± SD and statistical analysis was performed using the two‐sample Kolmogorov‐Smirnov test (at the 0.01 level).

	C‐arm	Halcyon MVCBCT HQ		Halcyon MVCBCT LD		Halcyon MVMV HQ		Halcyon MVMV LD	
	Value	Value	*P*	Value	*P*	Value	*P*	Value	*P*
Lumpectomy									
Max[Gy]	52.2 ± 0.4	52.2 ± 0.4	NS	52.2 ± 0.3	NS	52.1 ± 0.4	NS	52.1 ± 0.4	NS
Mean[Gy]	51.2 ± 0.5	51.2 ± 0.5	NS	51.0 ± 0.5	NS	50.9 ± 0.5	NS	50.9 ± 0.5	NS
D98%[Gy]	49.0 ± 3.0	49.1 ± 2.5	NS	49.1 ± 2.4	NS	48.9 ± 2.4	NS	48.7 ± 2.6	NS
V105%[%]	0.0 ± 0.0	0.0 ± 0.0	NS	0.0 ± 0.1	NS	0.0 ± 0.0	NS	0.0 ± 0.1	NS
H.I.	0.06 ± 0.06	0.06 ± 0.05	NS	0.06 ± 0.05	NS	0.06 ± 0.05	NS	0.06 ± 0.05	NS
Breast									
Max[Gy]	52.8 ± 0.3	52.6 ± 0.1	NS	52.7 ± 0.1	NS	52.7 ± 0.1	NS	52.6 ± 0.1	NS
Mean[Gy]	50.8 ± 0.3	50.9 ± 0.5	NS	50.9 ± 0.3	NS	50.8 ± 0.3	NS	50.8 ± 0.4	NS
D98%[Gy]	47.9 ± 0.6	48.3 ± 0.9	*P* < 0.01	48.3 ± 0.9	*P* < 0.01	48.2 ± 0.9	*P* < 0.01	48.3 ± 0.8	*P* < 0.01
V105%[%]	0.1 ± 0.2	0.0 ± 0.1	NS	0.0 ± 0.0	NS	0.0 ± 0.1	NS	0.0 ± 0.1	NS
H.I.	0.09 ± 0.01	0.08 ± 0.02	*P* < 0.01	0.08 ± 0.02	*P* < 0.01	0.08 ± 0.02	*P* < 0.01	0.08 ± 0.02	*P* < 0.01
MUs	376 ± 35	494 ± 51	*P* < 0.001	507 ± 54	*P* < 0.001	509 ± 55	*P* < 0.001	520 ± 62	*P* < 0.001

MVCBCT HQ, High‐quality megavoltage cone beam CT; MVCBCT LD, Low‐dose MVCBCT.

### 3.2 Doses to organs at risk

Tables 2 and 3 show the OARs DVH metrics for right‐sided and left‐sided treatments, respectively, including the *P* values from the Kolmogorov‐Smirnov test. Significant differences in Mean were observed for the CB, heart, and CL when comparing C‐arm and Halcyon plans regardless the imaging technique used and the highest increases were observed for the MVCBCT HQ plans.

**Table 2:** Dose to organs at risk in C‐arm plans and Halcyon plans for right‐sided treatments. Stated values indicate mean ± SD and statistical analysis was performed using the two‐sample Kolmogorov‐Smirnov test (at the 0.01 level).

	C‐arm	Halcyon MVCBCT HQ		Halcyon MVCBCT LD		Halcyon MVMV HQ		Halcyon MVMV LD	
	Value	Value	*P*	Value	*P*	Value	*P*	Value	*P*
Breast_L (contralateral)									
Max[Gy]	1.5 ± 0.4	3.3 ± 0.4	*P* < 0.001	2.1 ± 0.4	*P* < 0.001	1.6 ± 0.2	NS	1.4 ± 0.2	NS
Mean[Gy]	0.2 ± 0.2	1.1 ± 0.2	*P* < 0.001	0.6 ± 0.1	*P* < 0.001	0.7 ± 0.1	*P* < 0.001	0.4 ± 0.1	*P* < 0.001
V2Gy[cc]	0.1 ± 0.2	68.6 ± 35.8	*P* < 0.001	0.6 ± 1.4	NS	0.0 ± 0.0	NS	0.0 ± 0.0	NS
D0.1cc[Gy]	1.3 ± 0.3	2.9 ± 0.6	*P* < 0.001	1.9 ± 0.3	*P* < 0.001	1.5 ± 0.3	*P* < 0.01	1.2 ± 0.2	NS
Heart									
Max[Gy]	3.0 ± 1.0	5.3 ± 0.7	*P* < 0.001	4.2 ± 0.7	*P* < 0.01	4.4 ± 0.8	NS	3.6 ± 0.8	NS
Mean[Gy]	0.4 ± 0.3	2.0 ± 0.3	*P* < 0.001	1.2 ± 0.2	*P* < 0.001	1.0 ± 0.1	*P* < 0.001	0.8 ± 0.1	*P* < 0.001
V5Gy [%]	0.0 ± 0.0	0.1 ± 0.2	NS	0.0 ± 0.0	NS	0.0 ± 0.0	NS	0.0 ± 0.0	NS
Lung_R (ipsilateral)									
Mean[Gy]	5.4 ± 1.3	7.1 ± 1.3	*P* < 0.001	6.5 ± 1.4	*P* < 0.001	7.2 ± 1.2	*P* < 0.01	6.2 ± 1.4	NS
V5Gy [%]	19.2 ± 4.5	28.9 ± 4.7	*P* < 0.001	23.2 ± 4.2	*P* < 0.001	24.3 ± 3.4	NS	21.0 ± 4.3	NS
V10Gy [%]	11.6 ± 3.4	13.5 ± 3.5	NS	12.7 ± 3.5	NS	14.9 ± 2.7	NS	12.3 ± 3.5	NS
V20Gy [%]	8.3 ± 2.9	9.0 ± 3.1	NS	8.9 ± 3.1	NS	11.1 ± 2.4	NS	8.9 ± 3.1	NS
Lung_L (contralateral)									
Mean[Gy]	0.1 ± 0.1	0.8 ± 0.2	*P* < 0.001	0.4 ± 0.1	*P* < 0.001	0.3 ± 0.1	*P* < 0.001	0.2 ± 0.1	*P* < 0.001

MUs, monitor units; MVCBCT HQ, High‐quality megavoltage cone beam CT; MVCBCT LD, Low‐dose MVCBCT.

**Table 3:** Dose to organs at risk in C‐arm plans and Halcyon plans for left‐sided treatments. Stated values indicate mean ± SD and statistical analysis was performed using the two‐sample Kolmogorov‐Smirnov test (at the 0.01 level).

	C‐arm	Halcyon MVCBCT HQ		Halcyon MVCBCT LD		Halcyon MVMV HQ		Halcyon MVMV LD	
Breast_R (contralateral**)**									
Max[Gy]	1.3 ± 0.4	2.8 ± 0.4	*P* < 0.001	1.8 ± 0.3	*P* < 0.01	0.9 ± 0.1	NS	1.0 ± 0.2	NS
Mean[Gy]	0.1 ± 0.0	1.0 ± 0.3	*P* < 0.001	0.6 ± 0.2	*P* < 0.001	0.3 ± 0.1	*P* < 0.001	0.2 ± 0.1	*P* < 0.01
V2Gy[cc]	0.0 ± 0.0	21.5 ± 24.5	*P* < 0.001	0.1 ± 0.1	NS	0.0 ± 0.0	NS	0.0 ± 0.0	NS
D0.1cc[Gy]	1.0 ± 0.3	2.7 ± 0.4	*P* < 0.001	1.7 ± 0.2	*P* < 0.001	0.9 ± 0.1	NS	0.9 ± 0.2	NS
Heart									
Max[Gy]	11.1 ± 4.7	11.5 ± 4.1	NS	10.5 ± 4.0	NS	10.8 ± 6.2	NS	9.7 ± 4.0	NS
Mean[Gy]	0.9 ± 0.2	2.7 ± 0.3	*P* < 0.001	1.9 ± 0.2	*P* < 0.001	1.4 ± 0.3	*P* < 0.001	1.3 ± 0.2	*P* < 0.001
V5Gy[%]	0.7 ± 0.5	3.5 ± 1.6	*P* < 0.001	1.2 ± 0.7	NS	1.0 ± 1.0	NS	0.7 ± 0.5	NS
Lung_L (ipsilateral)									
Mean[Gy]	4.2 ± 1.2	5.7 ± 1.2	NS	5.1 ± 1.2	NS	4.9 ± 1.7	NS	4.7 ± 1.2	NS
V5Gy[%]	15.4 ± 4.4	23.6 ± 4.4	*P* < 0.001	19.1 ± 4.2	NS	18.7 ± 5.8	NS	17.0 ± 4.3	NS
V10Gy[%]	8.8 ± 3.6	10.3 ± 3.7	NS	9.5 ± 3.6	NS	9.6 ± 5.0	NS	9.1 ± 3.5	NS
V20Gy[%]	6.0 ± 2.8	6.3 ± 2.8	NS	6.2 ± 2.8	NS	6.2 ± 3.9	NS	6.1 ± 2.7	NS
Lung_R (contralateral)									
Mean[Gy]	0.0 ± 0.0	0.8 ± 0.2	*P* < 0.001	0.5 ± 0.2	*P* < 0.001	0.2 ± 0.0	*P* < 0.001	0.2 ± 0.0	*P* < 0.001

MVCBCT HQ, High‐quality megavoltage cone beam CT; MVCBCT LD, Low‐dose MVCBCT.

For the CB, the Mean increased from 0.1 ± 0.0 Gy and 0.2 ± 0.2 Gy on the C‐arm plans to 1.0 ± 0.3 Gy and 1.0 ± 0.3 Gy for right‐sided and left‐sided plans when the MVCBCT HQ was used (*P* < 0.001). The Max to the CB also had a significant increase for the MVCBCT HQ and MVCBT LD plans one both, right‐ and left‐sided treatments. Mean to the CB was 2.5 times higher for right‐sided lesions as compared with left‐sided lesions when the MV/MV technique was used. This can be explained considering that the gantry is fixed at 90° for the lateral MV regardless of the treatment side, irradiating the left side of the patient. Max to the heart was not significant different for left‐sided treatments regardless the imaging technique. For right‐sided treatments, significant differences were observed in heart Max for the MVCBCT HQ and LD plans. Heart Mean increased significantly for all the Halcyon plans at the *P* < 0.001 level. For the Mean IL, no significant differences were observed when comparing C‐arm and Halcyon plans for left‐sided treatments. For right‐sided treatments, a significant increase in IL Mean was observed for the MVCBCT HQ, MVCBCT LD and MV/MV HQ plans but not for the MV/MV LD plan. CL Mean also increased significantly, at the *P* < 0.001 level, for all the Halcyon plans.

### 3.3 Dose from imaging fields

**Figure 2:**
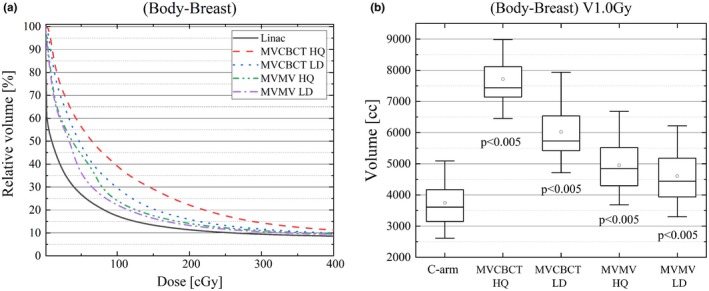
(a) Low‐dose region of the cumulative DVH of (Body‐Breast) over 25 fractions for the C‐arm and Halcyon plans. (b) Box plot showing the (Body‐Breast) V1.0Gy [cc], significant differences were observed when comparing C‐arm and Halcyon plans.

Figure 2(a) shows a representative cumulative DVH histogram for the (Body‐Breast) contour on the C‐arm and Halcyon plans over 25 fractions. The integral dose increases in Halcyon plans due to the imaging fields, with the dose difference being higher for the MVCBCT HQ plans. The subvolume of (Body‐Breast) receiving 1.0 Gy or more were 3746 ± 984 cc, 7718 ± 1016 cc, 6023 ± 1105 cc, 4954 ± 1117 cc, and 4605 ± 1133 cc for the C‐arm, MVCBCT HQ, MVCBCT LD, MVMV HQ, and MVMV LD plans, respectively [Fig. 2(b)].

**Figure 3:**
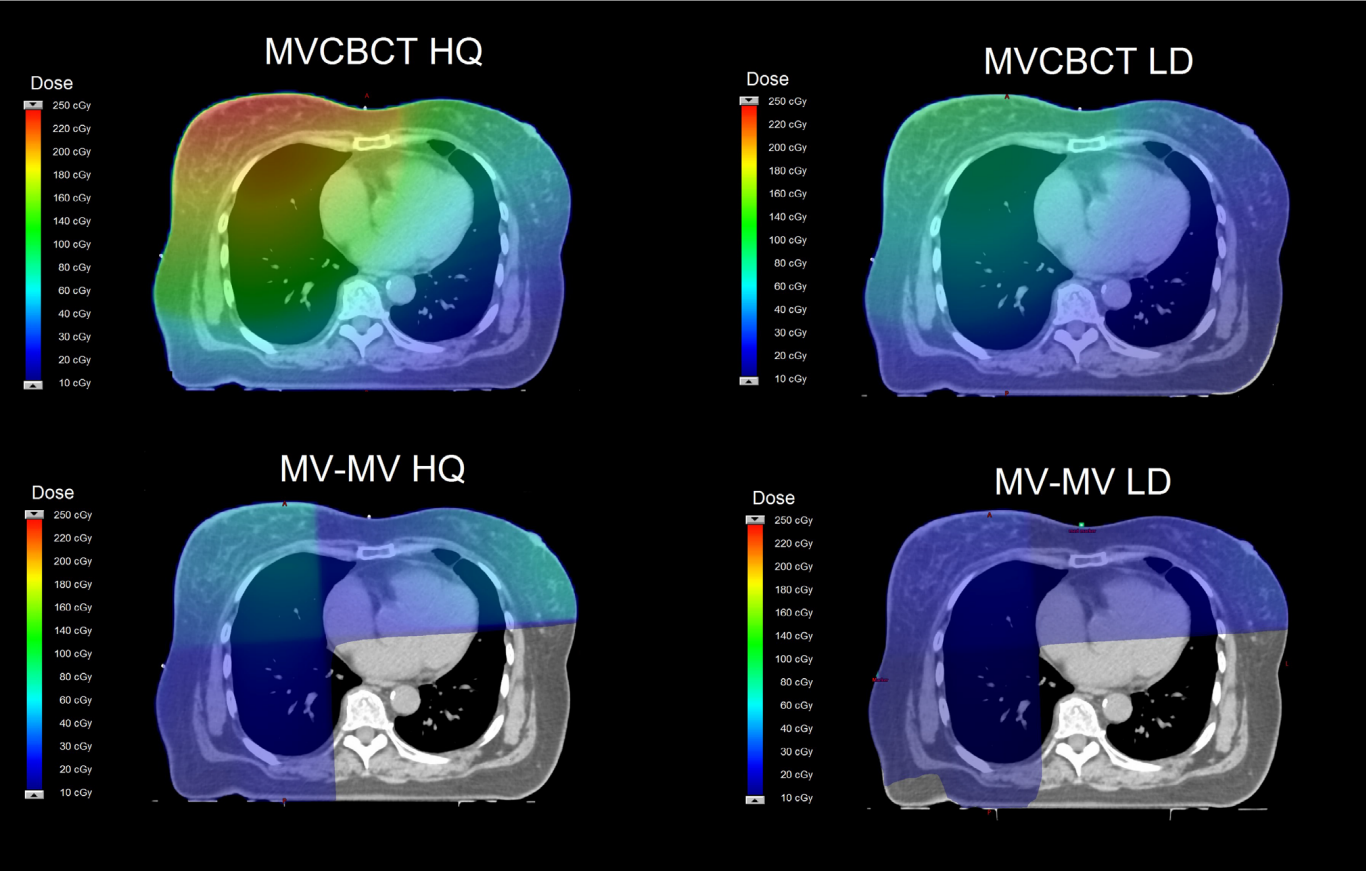
Dose distributions produced by the imaging fields on Halcyon for 25 fractions on a typical right‐sided breast treatment.

Figure 3 shows a typical dose distribution from each of the setup fields for 25 fractions on a right‐sided breast patient. For both MVCBCT fields, the OARs receiving more imaging dose were the CL and heart, while for the MV‐MV pairs, the IL and CB received the highest doses.

**Figure 4:**
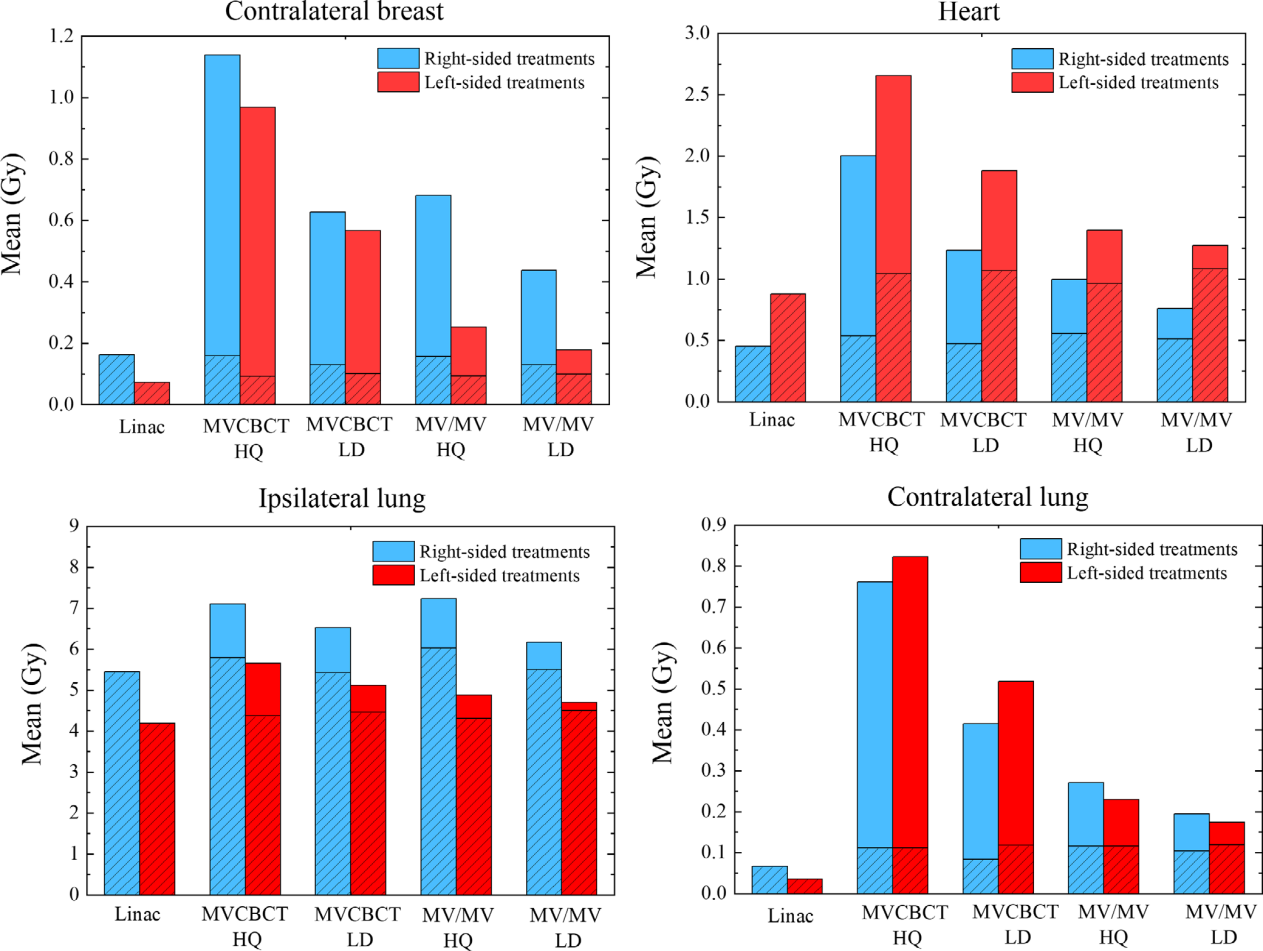
Organs at risks Mean for treatments using the C‐arm and Halcyon. The shaded area indicates the contribution to Mean from the treatment fields and the solid area indicates the contribution from the imaging fields.

Figure 4 shows the Mean for the OARs separating the contribution from the imaging fields and the contribution from the treatment fields. For the CB, the imaging fields are the dominating contribution to Mean regardless of the imaging technique and treatment side. For the heart, the dose from the MVCBCT HQ field is also higher than the dose associated with the treatment fields and the increase in Mean is approximately 1.5 Gy for left and right sided plans. For left‐sided lesions, the dose from the MV‐MV pairs is less than a half as compared with the dose for plans with right‐sided lesions. In contrast, the dominating contribution to the IL Mean is the treatment fields and the increase due to the imaging fields is less than 30% for any imaging technique.

## 4 DISCUSSION

### 4.1 Plan quality

Two factors may affect the plan quality between the C‐arm plans and the Halcyon plans: the MLCs and the difference on beam quality. Although the leaf width at isocenter is comparable, Halcyon’s MLC and the Millennium 120 MLC are different in terms of transmission, leaf speed, and dosimetric leaf gap as reported by Lim et al.^21^ Furthermore, Halcyon uses a FFF beam with different photon spectra than the flattened beam used for the C‐arm plans. The mean energy for photons at the central part of the flattened beam is higher as compared with FFF beams,^22^ and, as a result, small statistical differences on plan quality between FF and FFF beams have been reported.^23^, ^24^ However, for the plans investigated in this work, no significant differences were observed on the plan quality metrics between the C‐arm and Halcyon plans.

### 4.2 Setup fields

One limitation of the planning process is the unavailability of custom gantry angles for the MV images in Halcyon, where MV radiographs are taken at the predefined gantry angles of 0° and 90° without the possibility of the user to change that value. MV orthogonal pairs are unsuitable for setting up right‐sided patients, as the lateral beam provides the image of the opposite site of the patient. For this reason, the MVCBCT LD imaging technique is the most suitable for patients treated on Halcyon linacs without kV capabilities.

### 4.3 Dose to OARs

**Figure 5:**
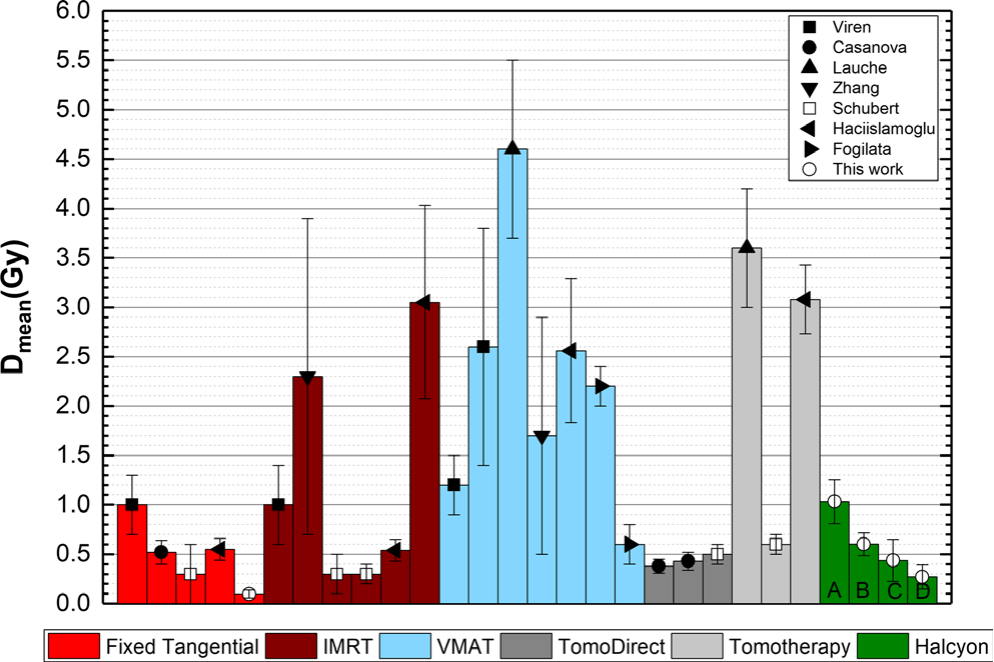
Comparison for CB Mean dose for different techniques and Halcyon. The labels on the Halcyon plans indicate the imaging technique used for setup: MVCBCT HD (a), MVCBCT LD (b), MV/MV HQ (c), and MV/MV LD (d). CB, contralateral breast.

Halcyon plans had higher dose values to the OARs than C‐arm plans for most of the metrics evaluated in this work. In particular higher doses to the CB are a concern as it has been reported that for women more than 40 yr old, mean doses to the CB higher than 1.0 Gy have a 2.5‐fold higher risk of developing a secondary cancer^6^, and this value is exceed only if the MVCBCT HQ technique is used. The mean dose to the CB is especially important for patients younger than 40 yr old or with mutations in the breast cancer susceptibility gene, BRCA2, as they represent the population with higher risk for secondary breast cancers. If the MVCBCT LD technique is used, as proposed in this work, the CB mean dose is 0.6 ± 0.1 Gy, which is within the range of values published in the literature for fixed tangential fields^6^, ^10^, ^12^, ^13^, ^18^ (from 0.3 to 1.0 Gy). For Halcyon plans, the mean dose to the CB is comparable or lower than other external beam modalities currently used in the clinical practice, such as TomoDirect^10^, ^11^, ^12^, ^13^ (from 0.3 to 0.6 Gy), Tomotherapy^10^, ^12^ (from 0.6 to 3.6 Gy), IMRT^10^, ^12^, ^15^, ^18^ (from 0.3 to 3.2 Gy), or VMAT^10^, ^11^, ^15^, ^17^, ^18^ (from 0.6 to 4.6 Gy), as shown in Fig. 5.

In the case of the heart, the probability of major coronary events increase linearly with the mean dose to the heart by 7.4% per Gy.^25^ Taylor et al,^26^ performed a systematic review of published doses to the heart from breast cancer radiation therapy and reported that for treatments that did not included the internal mammary nodes, the mean dose to the heart was 3.4 ± 0.2 Gy and 5.6 ± 0.4 Gy for tangential treatments and IMRT, respectively. In this study, for Halcyon plans using MVCBCT LD fields, the mean dose to the heart was 1.3 ± 0.1 Gy and 1.8 ± 0.3 Gy, for right‐sided and left‐sided targets, respectively. These values are also lower than the 4.0 Gy limit recommended in clinical trials such as the one performed by the Radiation Threapy Oncoloy Group (RTOG) 1005.^27^

### 4.4 Dose reduction options

As recommended by TG‐75 and following the ALARA principle, it is important to evaluate dose reduction techniques for IGRT. Some of the strategies can be implemented in the clinic by the physicist and some other will require modifications in the Halcyon workflow from the manufacturer. For example:

#### 4.4.1 Optimized imaging scheduling

To ensure that the setup of the patients is adequate and at the same time minimize the dose to OARs from the imaging fields, it is a common practice in many institutions, including ours, to have imaging schedules where port films are taken only once a week. This approach is not possible for Halcyon, as the imaging fields cannot be skipped at any fraction. An alternative to balance an adequate setup and also reduce the contribution of imaging dose, would be generating two plans, one including an MVCBCT LD and a second plan using MV‐MV ports. The proposed schedule consists on delivering the MVCBCT once a week and the MV/MV LD ports the rest of the fractions.

#### 4.4.2 Use of nonionizing imaging techniques

SGRT has been successfully used to setup and monitor linac‐based stereotactic radiosurgery and deep inspiration breath‐hold treatments.^28^, ^29^, ^30^ At our institution SGRT has been successfully implemented for setting up patients treated on the brain area with the Halcyon linac, reducing the residual rotational errors before the IGRT fields and the total treatment time.^31^ Surface‐guided radiotherapy could be used for breast patients in Halcyon for daily setup. To fully take advantage of this approach, the user should have the option to skip the imaging fields, however, this would require a site‐specific modification in the workflow by the manufacturer.

#### 4.4.3 Angled MV ports

**Figure 6:**
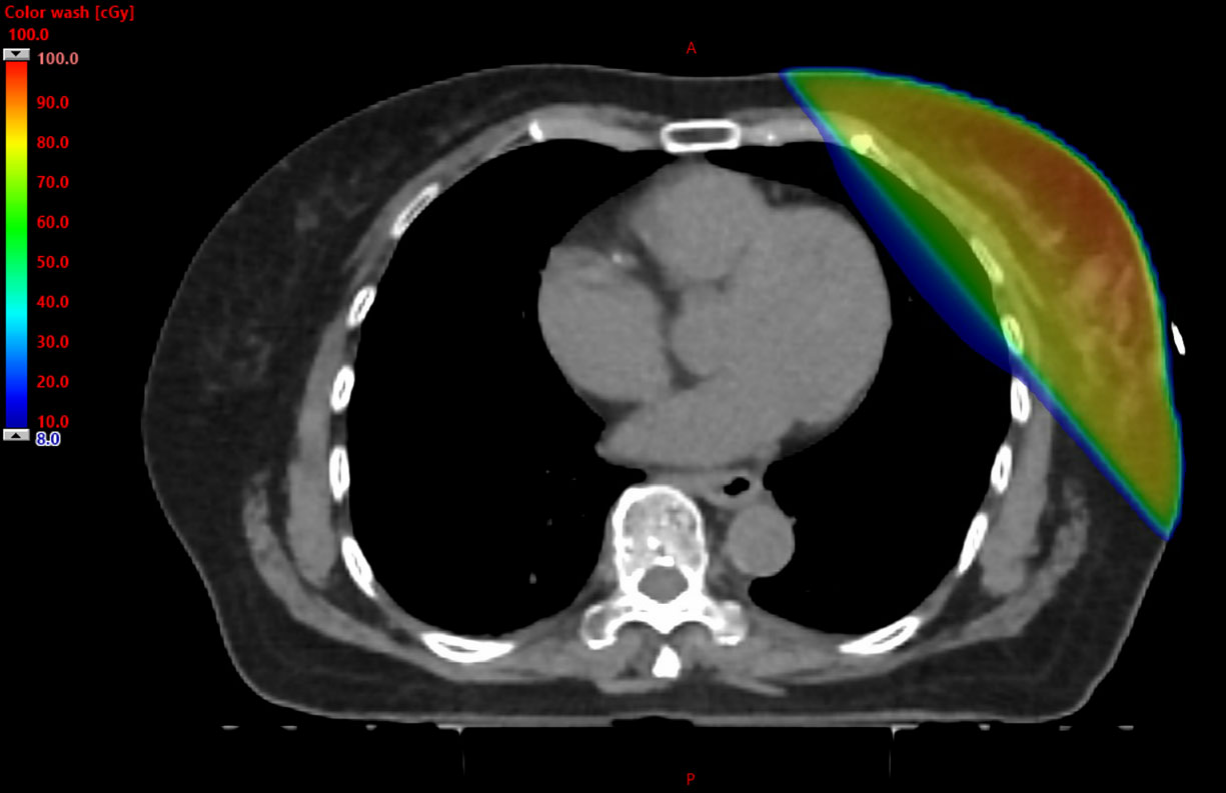
Dose distribution for a left‐sided target from a pair of rectangular MV fields at the treatment angles and delivering 2 MUs each. Angled MV ports are a widely used setup technique for C‐arm linacs as the breast is visible for alignment and the dose to the heart and CB are minimized. CB, contralateral breast; MUs, monitor units; MV, megavoltage.

Dose from the imaging fields may also be reduced with site‐specific modifications by the manufacturer, such as MV images at arbitrary angles. Tangential images at the treatment angles are part of the setup technique at our clinic using the C‐arm linac for breast patients as they allow the visualization of the breast and minimize the dose to the OARs, Fig. 6 shows the dose distribution for two rectangular MV fields delivering 2 MUs each. For 25 fractions the mean dose to the heart and CB associated with the imaging fields are 4.1 and 0.1 cGy, respectively.

## 5 CONCLUSION

This work is relevant for clinics with Halcyon linacs without the kVCBCT capability. For some of these clinics, Halcyon may be the only machine used for treatment and the performance for every treatment site needs to be investigated. Our work has shown that the Halcyon linac is suitable for treating breast patients with separations less than 22 cm, using the whole‐breast tangential beam technique, however, doses to OARs need to be considered when choosing the setup imaging technique. Geometry and beam related limitations should also be evaluated prior to treatment. Mean doses to OARs in Halcyon treatments using the MVCBCT LD technique are lower than mean doses reported from other techniques such as VMAT or IMRT, and can be further decreased by implementing imaging dose reduction techniques via optimized schedules or changing the workflow in Halcyon. Further research is necessary to investigate alternatives to tangential breast fields to treat patients with larger separations, boost the lumpectomy cavity and to implement the deep inspiration breath‐hold technique for left‐sided patients.

## CONFLICT OF INTEREST

We have no relevant conflict of interest to disclose.

## References

[1] DeSantis CE , Ma J , Goding Sauer A , Newman LA , Jemal A . Breast cancer statistics, 2017, racial disparity in mortality by state. CA Cancer J Clin. 2017;67:439–448. 28972651 10.3322/caac.21412

[2] Siegel RL , Miller KD , Jema A . Cancer Statistics, 2018. CA Cancer J Clin. 2018;68:7–30. 29313949 10.3322/caac.21442

[3] American Cancer Society . Breast Cancer Facts & Figures 2017–2018. Atlanta: American Cancer Society, Inc.; 2017.

[4] Murphy MJ , Balter J , Balter S , et al. The management of imaging dose during image‐guided radiotherapy: report of the AAPM task group 75. Med Phys. 2007;34:4041–4063. 17985650 10.1118/1.2775667

[5] Litière S , Werutsky G , Fentiman IS , et al. Breast conserving therapy versus mastectomy for stage I‐II breast cancer: 20 year follow‐up of the EORTC 10801 phase 3 randomized trial. Lancet Oncol. 2012;13:412–419. 22373563 10.1016/S1470-2045(12)70042-6

[6] Stovall M , Smith SA , Langholz BM , et al. Dose to the contralateral breast from radiotherapy and risk of second primary breast cancer in the WECARE study. Int J Radiat Oncol Biol Phys. 2008;72:1021–1030. 18556141 10.1016/j.ijrobp.2008.02.040 PMC3782859

[7] Sas‐Korczyńska B , Śladowska A , Rozwadowska‐Bogusz B , et al. Comparison between intensity modulated radiotherapy (IMRT) and 3D tangential beams technique used in patients with early‐stage breast cancer who received breast‐conserving therapy. Rep Pract Oncol Radiother. 2010;15:79–86. 24376929 10.1016/j.rpor.2010.06.002 PMC3863162

[8] Franco P , Zeverino M , Migliaccio F , et al. Intensity‐modulated adjuvant whole breast radiation delivered with static angle tomotherapy (TomoDirect): a prospective case series. J Cancer Res Clin Oncol. 2013;139:1927–1936. 24037488 10.1007/s00432-013-1515-0 PMC11824595

[9] Hashimoto H , Omura M , Matsui K , et al. Tangent field technique of TomoDirect improves dose distribution for whole‐breast irradiation. J App Clin Med Phys. 2015;16:225–232. 10.1120/jacmp.v16i3.5369 PMC5690132 26103495

[10] Haciislamoglu E , Colak F , Canyilmaz E , et al. Dosimetric comparison of left‐sided whole‐breast irradiation with 3DCRT, forward‐planned IMRT, inverse‐planned IMRT, helical tomotherapy, and volumetric arc therapy. Phys Med. 2015;31:360–367. 25733372 10.1016/j.ejmp.2015.02.005

[11] Lauche O , Kirova YM , Fenoglietto P , et al. Helical tomotherapy and volumetric modulated arc therapy: new therapeutic arms in the breast cancer radiotherapy. World J of Radiol. 2016;28:735. 10.4329/wjr.v8.i8.735 PMC5002504 27648167

[12] Schubert LK , Gondi V , Sengbusch E , et al. Dosimetric comparison of left‐sided whole breast irradiation with 3DCRT, forward‐planned IMRT, inverse‐planned IMRT, helical tomotherapy, and topotherapy. Radiother Oncol. 2011;100:241–246. 21316783 10.1016/j.radonc.2011.01.004

[13] Borca VC , Franco P , Catuzzo P , et al. Does TomoDirect 3DCRT represent a suitable option for post‐operative whole breast irradiation? A hypothesis‐generating pilot study. Radiat Oncol. 2012;7:211. 23241224 10.1186/1748-717X-7-211 PMC3547690

[14] Cozzi L , Fogliata A , Nicolini G , Bernier J . Clinical experience in breast irradiation with intensity modulated photon beams. Act Oncol. 2005;44:467–474. 10.1080/02841860510029879 16118080

[15] Zhang Q , Yu XL , Hu WG , et al. Dosimetric comparison for volumetric modulated arc therapy and intensity modulated radiotherapy on the left‐sided chest wall and internal mammary nodes irradiation in treating post‐mastectomy breast cancer. Radiol Oncol. 2015;49:91–98. 25810708 10.2478/raon-2014-0033 PMC4362613

[16] Tyran M , Mailleux H , Tallet A , et al. Volumetric‐modulated arc therapy for left‐sided breast cancer and all regional nodes improves target volumes coverage and reduces treatment time and doses to the heart and left coronary artery, compared with a field‐in‐field technique. J of Rad Res. 2015;56:927–937. 10.1093/jrr/rrv052 PMC4628222 26386255

[17] Fogliata A , Seppala J , Reggiori G , et al. Dosimetric trade‐offs in breast treatment with VMAT technique. Br J Radiol. 2017;90:20160701. 27885857 10.1259/bjr.20160701 PMC5685098

[18] Virén T , Heikkilä J , Myllyoja K , Koskela K , Lahtinen T , Seppälä J . Tangential volumetric modulated arc therapy technique for left‐sided breast cancer radiotherapy. Radiat Oncol. 2015;10:79. 25888866 10.1186/s13014-015-0392-x PMC4404692

[19] Mayo C , Moran JM , Bosch W , et al. American association of physicists in medicine task group 263: standardizing nomenclatures in radiation oncology. Int J Radiat Oncol Biol Phys. 2018;100:1057–1066. 29485047 10.1016/j.ijrobp.2017.12.013 PMC7437157

[20] International Commission on Radiation Units and Measurements (ICRU) . Prescribing, recording and reporting photon‐beam intensity modulated radiation therapy (IMRT), ICRU Report 83. J ICRU. 2010; 10:27–38.

[21] Lim TY , Dragojević I , Hoffman D , Flores‐Martinez E , Kim GY . Characterization of the Halcyon^TM^ multileaf collimator system. J Appl Clin Med Phys. 2019;20:106–114. 30889312 10.1002/acm2.12568 PMC6448159

[22] Dalaryd M , Kragl G , Ceberg C , et al. A Monte Carlo study of a flattening filter‐free linear accelerator verified with measurements. Phys Med Biol. 2010;55:7333. 21081829 10.1088/0031-9155/55/23/010

[23] Hansen CR , Bertelsen A , Riis HL , et al. Plan quality and delivery accuracy of flattening filter free beam for SBRT lung treatments. Act Oncol. 2015;54:422–427. 10.3109/0284186X.2014.956184 25238280

[24] Nicolini G , Ghosh‐Laskar S , Shrivastava SK , et al. Volumetric modulation arc radiotherapy with flattening filter‐free beams compared with static gantry IMRT and 3D conformal radiotherapy for advanced esophageal cancer: a feasibility study. Int J Radiat Oncol Biol Phys. 2012;84:553–560. 22386376 10.1016/j.ijrobp.2011.12.041

[25] Darby SC , Ewertz M , McGale P , et al. Risk of ischemic heart disease in women after radiotherapy for breast cancer. N Engl J Med. 2013;368:987–998. 23484825 10.1056/NEJMoa1209825

[26] Taylor CW , Wang Z , Macaulay E , Jagsi R , Duane F , Darby SC . Exposure of the heart in breast cancer radiation therapy: a systematic review of heart doses published during 2003 to 2013. Int J Radiat Oncol Biol Phys. 2015;93:845–853. 26530753 10.1016/j.ijrobp.2015.07.2292

[27] Vicini F , Freedman GM , White JR . A phase III trial of accelerated whole breast irradiation with hypofractionation plus concurrent boost versus standard whole breast irradiation plus sequential boost for early‐stage breast cancer. RTOG. 1005.

[28] Cervino LI , Gupta S , Rose MA , Yashar C , Jiang SB . Using surface imaging and visual coaching to improve the reproducibility and stability of deep‐inspiration breath hold for left‐breast‐cancer radiotherapy. Phys Med Biol. 2009;54:6853. 19864703 10.1088/0031-9155/54/22/007

[29] Cervino LI , Pawlicki T , Lawson JD , Jiang SB . Frame‐less and mask‐less cranial stereotactic radiosurgery: a feasibility study. Phys Med Biol. 2010;55:1863. 20224158 10.1088/0031-9155/55/7/005

[30] Cerviño LI , Detorie N , Taylor M , Lawson JD , Harry T , Murphy KT . Initial clinical experience with a frameless and maskless stereotactic radiosurgery treatment. Pract Radiat Oncol. 2011;2:54–62. 24674037 10.1016/j.prro.2011.04.005

[31] Flores‐Martinez E , Cervino L , Pawlicki T , Kim G . WE‐C930‐GePD‐F5‐03: improved setup and workflow for brain treatments on the Halcyon Linac by using surface guided radiation therapy. Med Phys. 2018;45:E592.

